# Monitoring and Discovery for Self-Organized Network Management in Virtualized and Software Defined Networks

**DOI:** 10.3390/s17040731

**Published:** 2017-03-31

**Authors:** Ángel Leonardo Valdivieso Caraguay, Luis Javier García Villalba

**Affiliations:** Group of Analysis, Security and Systems (GASS), Department of Software Engineering and Artificial Intelligence (DISIA), Faculty of Information Technology and Computer Science, Office 431, Universidad Complutense de Madrid (UCM), Calle Profesor José García Santesmases, 9, Ciudad Universitaria, 28040 Madrid, Spain; angevald@ucm.es

**Keywords:** 5G, Monitoring, NFV, SDN

## Abstract

This paper presents the Monitoring and Discovery Framework of the Self-Organized Network Management in Virtualized and Software Defined Networks SELFNET project. This design takes into account the scalability and flexibility requirements needed by 5G infrastructures. In this context, the present framework focuses on gathering and storing the information (low-level metrics) related to physical and virtual devices, cloud environments, flow metrics, SDN traffic and sensors. Similarly, it provides the monitoring data as a generic information source in order to allow the correlation and aggregation tasks. Our design enables the collection and storing of information provided by all the underlying SELFNET sublayers, including the dynamically onboarded and instantiated SDN/NFV Apps, also known as SELFNET sensors.

## 1. Introduction

The management and customization of network services have been limited by the rigidity of traditional network architectures and increasing both capital and operational expenditures. Actually, the resolution of common traditional network problems, such as link failures, security attack, Quality of Service (QoS) or Quality of experience (QoE) degradation, bottlenecks, among others, requires the direct involvement of network operators. The manual re-configuration of the existing equipment or even the installation of new equipment (router, NATs, firewalls) compromises the normal operation of the network and causes the disruption of the Service Level Agreements (SLAs). Similarly, the creation of innovative value-added services is limited by the closed and proprietary hardware/software, and in some cases, all infrastructure may belong to the same provider.

Those limitations make traditional network architectures unfeasible to meet the requirements of today’s users, enterprises and carriers. The solution proposed to solve these challenges is following the advances reached by computing, where developers can create their own applications using a high level programming language. The programs can be executed with different equipment thanks to the abstractions of resources provided by the Operating Systems. In this context, Software Defined Networking (SDN) and Network Function Virtualization (NFV) appears as a promising strategy to reach those objectives. SDN proposes the decoupling of data and control planes in network devices enabling their independent development and a centralized view of the network. NFV promotes the migration from typical network equipment (DPI, firewall, load balancers) to a software packages or network functions (NF) that can be instantiated in a virtualized infrastructure. Both architectures are complementary and potentially could be integrated to provide an open network environment for developers.

Furthermore, the exponential growth of mobile devices and content together with the advent of cloud services bring additional challenges to operators and service providers. A radical decrease of integrated network management operations without negatively affecting the QoS/QoE and security is required. Similarly, a new model that integrates the access and management of mobile resources is promoted. The future 5G architecture is expected to expose not only typical mobile broadband but also a heterogeneous, simplified and unified control [1]. The network management expenditures can be reduced through automation of operations. In this context, a scalable management framework that includes data mining, pattern recognition, learning algorithms to reduce operation expenditures is challenging.

The present paper comprises the following contributions. Firstly, the Self-Organized Network Management in Virtualized and Software Defined Networks Project (SELFNET) [2] is described. SELFNET uses SDN/NFV principles to provide smart autonomic management of network functions in order to resolve network problems or improve the QoS/QoE. SELFNET integrates the self-management paradigm with the use of data mining, learning algorithms, pattern recognition to identify the network behaviour and including 5G mobile architectures. The SELFNET architecture is composed of well defined layers: Infrastructure, Virtualized Network, SON Control, SON Autonomic and Access Layer.

Secondly, the present paper proposes the SELFNET Monitoring and Discovery Framework, as part of the Monitor and Analyzer sublayer of the SON Autonomic Layer. The SELFNET Monitoring and Discovery Framework lay the SDN/NFV foundations to provide a contextaware model based on a customizable set of low-level metrics. In contrast with traditional network monitoring schemas, the present framework integrates different sources within a single customizable system. In this way, it is able to organize the collected information according to different criteria such as virtual instances by tenant, metrics by virtual instances, physical devices by network location, physical metrics, flow statistics by device and metrics by sensor. Similarly, the proposed framework will facilitate not only the querying process of gathered information but also the management of large amounts of data from heterogeneous data sources. In this way, the data of each of these layers can be correlated to provide enhanced information of the network status such as the relation between virtual instances and their respective physical device, the physical and virtual instances where a sensor is running, information related to LTE devices, edges and locations. Through it, the administrator is able to establish policies based on high level paradigms or Health of Network (HoN) metrics. This resulted in a significant reduction in the costs and improvements to data quality and timeliness.

The rest of the paper is outlined as follows: Section 2 describes SDN, NFV and the related works focused on management and monitoring. Then, Section 3 explains the SELFNET Framework. Section 4 defines the Monitoring and Discovery Framework. Section 5 describes the sensor workflows (onboarding, instantiation and monitoring) following by each component of this framework. Section 6 describes the implementation of the framework. Finally, Section 6 presents the conclusions and future work.

## 2. Software Defined Networking and Network Function Virtualization

### 2.1. Software Defined Networking

Software Defined Networking (SDN) was born due to the lack of flexibility and programmability issues of the traditional deployments. The updating/change in network behaviour requires the manual reconfiguration (one by one) of the equipment. The inclusion of new network services is delayed due the large standardization process to introduce a new technology or standard [3]. SDN [4] proposes the separation between data and control plane in network devices enabling their independent development. It also proposes the logical centralized control of the network devices by means of an element known as controller. This element has a whole view of the network status and can manage all physical devices. The controller abstracts the network resources and provides an API that can be used by the network administrator to develop the network services.

**Figure 1:**
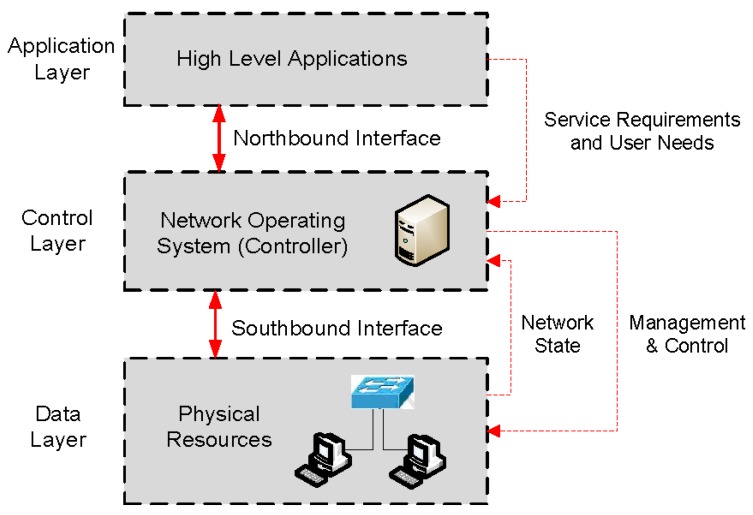
SDN Architecture.

SDN is promoted by the Open Networking Foundation (ONF) [5], which is a non-profit organization supported by the network operators and service providers. The SDN architecture defines three layers: Data layer, Control layer and Application layer in order to expose the network resources to external applications, as shown in Figure 1.

➢ Data layer: this layer exposes the resources of hardware devices (switches or routers) towards the control layer. The communication between data and control layer is done by the southbound interface.
➢ Control layer: the controller manages the network devices based on its policies or in high level applications. The API between control and application layer is the northbound interface.
➢ Application layer: this layer allows the development of high level applications in order to customize easily the network behaviour.

The main southbound interface is the OpenFlow protocol [6]. In contrast to well-known protocols based on traditional network architectures (NetFlow, sFlow, SNMP), OpenFlow is the first standard interface for the communication between data and control planes in SDN architecture. In this context, an OpenFlow switch takes into account the common set of functions of traditional switches in order to forward the information based on the controller instructions. The control plane (controller) sends OpenFlow messages to modify the rules in the switch tables and control the handling of the incoming packets.

### 2.2. Network Function Virtualization

The term “Virtualization” is not a new concept. The virtualization refers to the abstraction of the logical resources from the physical resources, creating multiple logical instances over the same physical infrastructure [7]. The virtualization has reached different technical domains: virtualization of operating systems, hardware platforms, storage capacities and networks. In the networking field, the virtualization enables the sharing of multiple virtual networks (VNs) over a physical network simultaneously. However, the virtualization principles have also been extended to other functionalities of networks.

**Figure 2:**
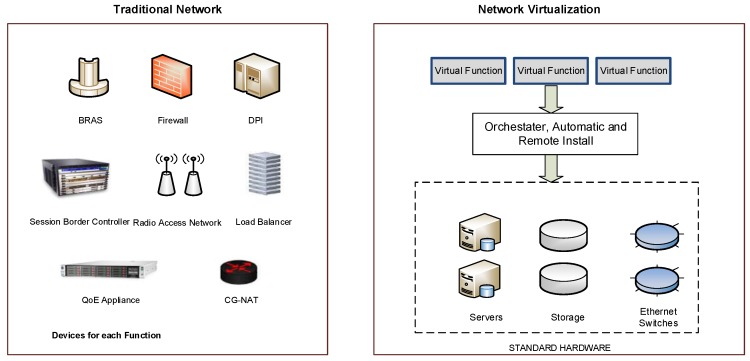
Traditional Network Functions vs. Virtual Network Functions.

Nowadays, the telecom providers invest a lot of money updating or installing new network functions (NFs) or appliances. Often, the appliances (e.g., firewall, DPI) are deployed in proprietary hardware or private software, and consequently, cannot be reused or modified by other service providers. Moreover, the rigidity and complexity of network deployments reduce the customization capabilities of the services. In this context, the Network Function Virtualization (NFV) [8,9] proposes the transferring of the Network Functions NF (routing, firewall, deep packet inspection DPI, gateway) towards virtual software-based applications, which are executed in IT platforms (servers, switches and storage). This new vision of IT services provides a major flexibility/scalability and facilitates the development of new Virtual Network Functions (VNFs), while decreasing costs. Figure 2 describes the differences between NFV and traditional architectures.

**Figure 3:**
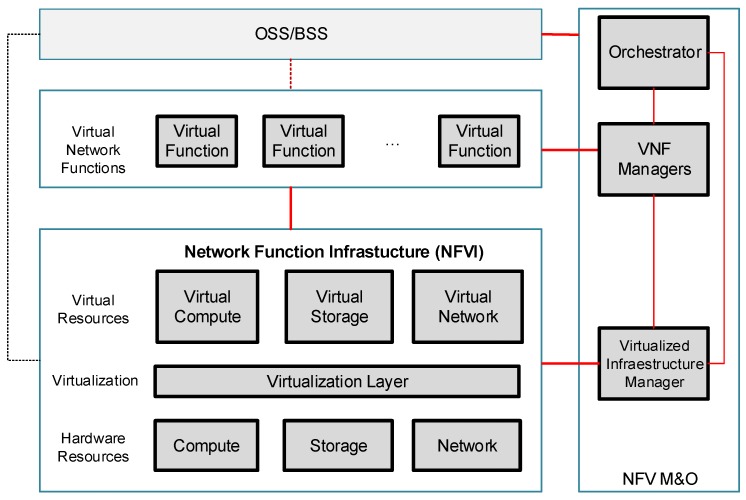
NFV architecture.

The European Telecommunications Standards Institute (ETSI) has released the first NFV specification [10]. The NFV architecture is composed of three main modules: NFV Infrastructure (NFVI), NFV and NFV Management and Orchestration (NFV M&O), as is shown in Figure 3.

The NFV components are described below:

➢ NFVI: It represents the hardware and software resources of the system where the NFV concept are applied. Computing, storage and networking resources are included. The virtualization layer abstracts the different resources and enables the isolation and independence of virtual compute, virtual storage and virtual network for different tenants.
➢ VNF: It represents a virtual NF instance that runs over the NFVI. A virtual NF could be deployed in a single virtual machine (VM) as well as over multiple VMs (different components in each virtual machine).
➢ NFV M&O: The main function is the orchestration and management of the VNF and NFVI ensuring the optimal and effective operation of the VNFs in the available infrastructure. The principal NFV M&O components are: the orchestrator, the VNF manager and the Virtualized Infrastructure Manager (VIM). The VIM uses a resource inventory to control the resources availability, which guarantees the provisioning of services.

### 2.3. Related Works

The SDN and NFV paradigms have been applied in several research areas. However, the present work focuses on two main research topics: network management and monitoring. In the first area, the use of SDN/NFV in the development of novel management architectures over virtualized environments has gained the interest of several consortiums formed by operators, academia and service providers. In Table 1, relevant SDN/NFV-based management projects are described. These projects involve different application scenarios. However, the provisioning of autonomic management operations capable to provide a scalable, extensible and smart network management is at the beginning stage of research.

Regarding the second research area, the monitoring on SDN and NFV plays a major role to the good operation of the network. The decisions taken by the different services depends on the high-speed and the accuracy of the information provided by monitoring services. In this context, the work proposed in [16] uses SDN to virtualize a switch to monitor traffic without the need of port mirroring. However, the system does not collect additional metrics at different levels (e.g., virtual, sensors). Similarly, NetAnlytics [17] can efficiently monitor data plane packet flows and uses NFV to instantiate the network functions. Nevertheless, the collected data is focused on flow metrics and does not provide customization of heterogeneous sources.

The monitoring framework presented on [18] enables the Virtual Infrastructure Manager monitoring. It includes different agents able to collect data from VNFs. However, the monitoring framework is considered to be an internal functional block of the VIM and does not include additional sources (e.g., physical metrics) and customization of tasks (e.g., aggregation). In [19], the authors propose a reference framework for traffic engineering for SDN networks. The framework is composed of traffic measurement (network level) and traffic management (QoS, load balancing). However, the proposal does not include the customization capabilities provided by NFV engine.

## 3. Self-Organized Network Management for SDN/NFV (SELFNET)

The SELFNET H2020 project focuses on design and implement an autonomic network management framework to provide Self-Organizing Network (SON) capabilities in new 5G mobile network infrastructures. By automatically detecting and mitigating a range of common network problems, currently manually addressed by network administrators, SELFNET aims to provide a framework that can significantly reduce operational costs and consequently improve the user experience [2,20]. By exploring the integration of novel technologies such as SDN, NFV, SON, Cloud Computing, Artificial Intelligence, QoS/QoE and next generation of networking, SELFNET will provide a scalable, extensible and smart network management system. The framework will assist network operators to perform key management tasks such as automatic deployment of SDN/NFV applications that provide automated network monitoring and autonomic network maintenance delivered by defining high-level tactical measures and enabling autonomic corrective and preventive actions to mitigate existing or potential network problems.

**Figure 4:**
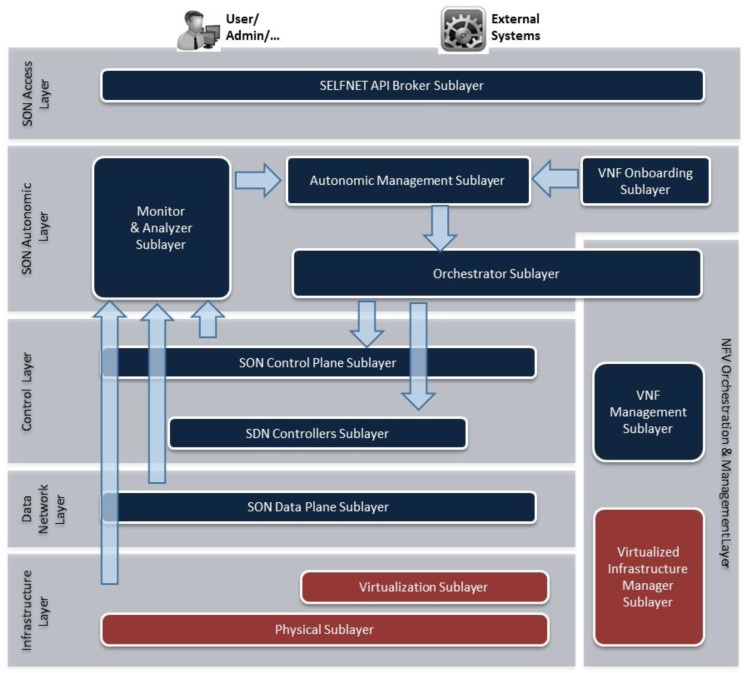
SELFNET Architecture Overview.

SELFNET addresses three major network management concerns by providing self-protection capabilities against distributed network attacks, self-healing capabilities against network failures, and self-optimization features to improve dynamically the performance of the network and the QoE of the users. The facilities provided by SELFNET will provide the foundations for delivering some of the 5G requirements defined by 5G-PPP consortium. In this context, the Figure 4 illustrates the architecture of the SELFNET framework. The architecture is based on five differentiated layers with the following logical scopes: Infrastructure Layer, Virtualized Network Layer, SON Control Layer, SON Autonomic Layer, NFV Orchestration & Management Layer, SON Access Layer.

### 3.1. Infrastructure Layer

This layer provides the resources required for the instantiation of virtual functions (Compute, Network and Storage) and supports the mechanisms for that instantiation. It represents the NFVI as defined by the ETSI NFV terminology [10]. In order to achieve its functionality, two sublayers will be defined: Physical Sublayer and Virtualization Sublayer.

➢ Physical Sublayer: It includes the physical resources required to provide computation, networking and storage capabilities over bare metal. Since SELFNET is also designed to include 5G networks, the physical elements follows a mobile edge architecture in which operators can deploy the operational and management services. The framework follows the Mobile Edge Computing (MEC) architecture proposed by ETSI [21] where the edge nodes are geographically separated from the data centre.
➢ Virtualization Sublayer: It enables the sharing of the available resources between different users or services. It offers several advantages, such as the isolation, reliability, adaptability and control of resources. However, the main disadvantage includes the poor performance in the virtualization tasks. Regarding this research topic, recent advances on virtualization technologies have reached the expectations on 5G infrastructures on the performance of virtualized workload with intensive Input/Output (I/O) [22]. In other words, the performance penalty of using virtualization can be considered as negligible for modern devices. In SELFNET, the virtualization sublayers includes the use of virtual switches used to connect Virtual Machines allocated on the physical resources.

### 3.2. Data Network Layer

In this layer, the different functionalities of the networks functions are located and interconnected in a designed topology. The NFs include the instances required for the normal operation of the virtual infrastructure and those created by SELFNET as part of the SON functionalities. The Data Network Layer also provides multi-tenancy support [23]. Multitenancy enables the sharing of resources among different tenants, each with their own administrative domain and business requirements. In this context, the term Software Defined Networking plays an important role. In traditional architectures, the network elements is composed by a specialized hardware in the packet processing (data plane), and over the hardware works an operating system (e.g., Linux) that receives information from the hardware and executes a software application (control plane). Instead, SELFNET follows the SDN principles and proposes the separation and decoupling of Data Network Layer and SON Control Layer. Additionally, open interfaces between them are established. Therefore, OpenFlow [6] is the most widely used protocol for exchanging information between data and control planes. The advantage of OpenFlow is the use of available elements and features of traditional network hardware. OpenFlow opens up these elements with a firmware update avoiding a complete change of hardware. In this way, OpenFlow acts as a standard way of conveying flow-table information to the network devices, so these can be controlled externally.

### 3.3. SON Control Layer

This layer includes the elements responsible of collecting data from different virtualized sources (SON Sensors) and the functions that execute actions into the network (SON Actuators). The SON Sensors and SON Actuators are controlled by the SON Autonomic Layer, which provides network intelligence. Similarly, the SON Control Layer deals with the control plane in SDN architectures. In other words, it translates autonomic network-wide policies into specific network element configurations. This layer is composed of two sublayers: SDN Controller Sublayer and SON Control Plane Sublayer.

- SDN Controller Sublayer: It implements a logically centralized controller (e.g., SDN control plane) and provides the governance of the network elements and the control of the network functions. The SDN controller uses the information of the network behaviour and enforces the execution of the rules in the network elements. In this way, the traffic passing through such network elements can be dynamically modified. For this purpose, the SDN controller uses a well-defined API and standardized forwarding instruction set (e.g., OpenFlow).
- SON Control Plane Sublayer: It instantiates the different network functions (NF) in the virtualized infrastructure. In SELFNET architecture, which aims to provide self-organized capabilities, there are two types of NFs: SON Sensors and SON Actuators. The SON Sensors collect data related to network activities. The collected information includes metrics related to global traffic (e.g., link status, bandwidth) or specific metrics (e.g., DPI, QoS on a video streaming of a specific data flow). The SON Actuators execute a specific set of actions on the traffic circulating in the network. The actions depend on the application developed by the service providers. For instance, if the system detects a DDoS attack, a SON actuator can automatically block the specific attack source. If the system detects a QoS degradation, another SON Actuator can optimize the network flow increasing the priority or bandwidth.

### 3.4. SON Autonomic Layer

This layer is responsible of providing the network intelligence. The information collected from sensors is used to diagnose the network situation. Then, the actions to accomplish the systems goals are determined and executed. The main components of SON Autonomic Layer are described as follows.

#### 3.4.1. Monitor and Analyzer

This sublayer collects the information provided by sensors. Then, this information is aggregated and correlated in order to extract the relevant information. The analyser uses the relevant information to detect network situations (botnet detected, QoS/QoE degradation, DDoS attack, link failure). The whole process is organized in three steps: Monitoring and Discovery, Aggregation and Correlation and Analyzer.

➢ Monitoring and Discovery. It collects the data sent by the SON Sensors. For this purpose, when a new Sensor is instantiated, it receives the notification and instantiation details and establishes a connection in order to receive the corresponding metrics. Moreover, it also receives the information provided by the physical and virtual sublayers. Then, the information is stored in a database in order to be processed by upper layers.
➢ Aggregation and Correlation. It performs the correlation and aggregation of the information stored in the monitoring and discovery database. This process involves additional actions, such as the data normalization, verification and removal of redundant information. At the end of this stage, only relevant information will be processed by the Analyzer module.
➢ Analyzer. Its main purpose is the comprehensive analysis of the relevant information provided by Aggregation and Correlation. The analysis also includes the prediction of future network problems. The network problems are known as Health of Network (HoN) due the global vision or system-level scope of the analysis. For this purpose, it takes advantage of several prediction, pattern recognition algorithms and big data techniques. The trending and predicted values for the metrics enable the application of proactive and reactive actions in the system. At the end of this stage, the network events are sent to the autonomic manager in order to establish the corresponding actions in the network.

#### 3.4.2. VNF Onboarding

It acts as a repository of the different network functions NFs. In this sublayer, the available network functions are stored and their capabilities are disseminated to the other sublayers. Similarly, the service providers can design, create and update their own applications. In this context, the encapsulation of NFs follows the recommendations of the ETSI MANO framework for the NFV [24]. Consequently, the VNF Manager (VNFM) is the key component for the lifecycle of SON sensors and actuators. The VNFM lifecycle exposes a common set of primitives for the automated instantiation, configuration, re-configuration and termination of the different VNFs. A common API enables service providers the easy design and development of their solutions. Once a solution (NFs) is onboarded, the autonomic manager can use their capabilities to provide the new service (sensor/actuator).

#### 3.4.3. Autonomic Manager

It uses different algorithms to diagnose the root cause of a network problem in terms of the HoN metrics provided by the Analyzer. Once the cause is detected, the autonomic manager uses the available NFs provided by the VNF onboarding to decide the best reaction strategy or a countermeasure (e.g., deploy a new balancer, firewall or DPI). Then, the taken actions are sent to the NFV Orchestration and Management Layer. The related tasks are organized in three well defined modules.

➢ Diagnoser. It diagnoses the root cause of the network situations notified by the analyzer. For this purpose, it uses the information available on Monitor & Analyzer sublayer (topology, sensor data, HoN metrics) and takes advantage of stochastic algorithms [25,26], artificial intelligence [27,28], data mining [29] to estimate the location of the source of the problem. Then, the root cause is notified to the Decision Maker. Because Diagnoser follows the extensibility and customization capabilities defined in 5G networks [1], the architecture enables the existence of a dataset of several algorithms. Depending of the complexity of the situation, SELFNET defines three different diagnosis processing chains: TAL-based diagnosis, machine learning diagnosis and offline diagnosis. More information about diagnose component can be found in [30,31].
➢ Decision Maker. It takes the incoming diagnosis information and decides a set of reactive and proactive actions to be taken into the network in order to avoid the detected and emerging network problems, respectively. Similarly, it also takes advantage of the integration of artificial intelligence algorithms to determine the responses or tactics to be taken. The taken decisions are notified to the action enforcer.
➢ Action Enforcer. It provides a consistent and coherent scheduled set of actions to be taken in the infrastructure. In other words, it validates, organizes and refines the tactics to avoid conflicts, duplications and nonsense order of actions. At the end of this stage, a high level description of the location, type of SELFNET SON Actuators, related configuration parameters are transferred to the orchestrator.

### 3.5. NFV Orchestration and Management Layer

This layer is responsible for the control and chaining of the different NFs in the virtualized infrastructure. The architecture follows the ETSI MANO [24] recommendations and, consequently, it is composed of: Orchestration, VNF Management and Virtualized Infrastructure Management (VIM). As described in Section 3.4.2, the VNF Management operations are partially developed in the VNF Onboarding. The other operations are described as follows:

➢ NFV Management and Orchestration. It is responsible for receiving the set of actions of the Autonomic Manager and orchestrate the network functions in the available virtual resources. The coordination and schedule of the enforcement of different actions is executed by the interaction with the virtual infrastructure manager.
➢ Virtual Infrastructure Manager VIM. It is responsible for organizing and providing the virtual resources for the instantiation of the different network functions. The VIM interacts with the physical and virtual infrastructure to ensure the availability of resources and perform the automatic deployment of services.

### 3.6. SON Access Layer

This layer provides an appealing and intuitive interface that enables different monitor and operation capabilities depending on the authorized users. In this way, the users can check the current health status of the SELFNET operations. Similarly, the Access API lists the SON Sensor and Actuator currently deployed in SELFNET as well as the loggings and messages in order to enable a wider view of the SELFNET status. This interface is used by external actors such as Business Support Systems (BSS) or Operational Support Systems (OSS). As described in the previous sections, SELFNET aims to be an independent and autonomous solution that acts mitigating or solving network problems without any actions from real users. In this way, the SON Access Layer also provides users the validation of the actions taken by the SELFNET autonomous system.

## 4. SELFNET Monitoring and Discovery

**Figure 5:**
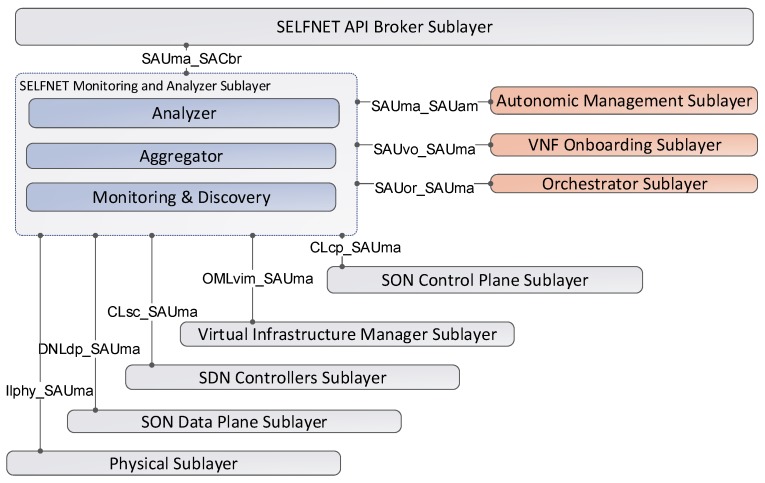
Monitor and Analyzer Sublayer Interfaces.

One of the main challenges of the SELFNET infrastructure is the monitoring and discovery of the different metrics generated by the underlying virtualized infrastructure. The metrics collected can include low-level metrics, Key Performance Indicators (KPI) and Health of Network Metrics (HoN) in order to have a complete knowledge about the network status. However, unlike the traditional monitoring solutions, where the monitoring nodes and information provided are static, the SELFNET sensors are virtualized network functions that can be dynamically allocated in different sections of the network. Moreover, the SELFNET Monitoring and Discovery Framework should be able to monitor a large amount of low-level metrics from several data sources. In this context, Figure 5 shows the eight interfaces used for either collecting metrics or sending the results of analysis tasks.

The SELFNET Monitor and Analyzer interacts with other sublayers through different APIs. The Table 2 describes the interface name, source, destination and the provided information. On one hand, several sources send their corresponding information to be processed and analysed. On the other hand, the output of the analysis is sent to the autonomic sublayer and the status of the sublayer is delivered to the broker sublayer. In order to achieve the functionality established by the SELFNET architecture, the proposed framework takes into account different design principles and methodologies, as summarized in Table 3. A detailed description of the SELFNET System Requirements can be found in [30].

### 4.1. High Level Architecture

The main goal of Monitoring and Analyzer sublayer is to provide a consistent set of components to analyze network status by the proper use of the monitoring information gathered from the running network infrastructure. With this objective in mind, the Monitoring and Analyzer Sublayer was divided in three sections: Monitoring and Discovery, Aggregation and Analyzer. The Analyzer Framework relies on Health of Network (HoN) metrics to determine network status. The outcomes of this framework are sent to the Autonomic Management Sublayer. HoN metrics, in turn, are derived from the aggregated or correlated low-level metrics and events provided by the Aggregation Framework. Low-level metrics are collected in the Monitoring and Discovery Framework.

**Figure 6:**
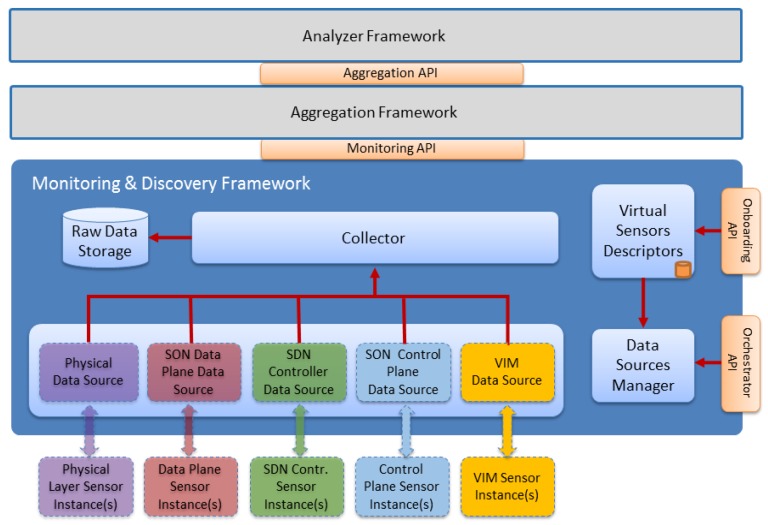
Monitoring and Discovery Framework Architecture.

The whole architecture is divided in functional blocks, as shown in the Figure 6. In the bottom part, the underlying data sources are located. The Monitoring and Discovery framework introduces the concept of Data Source as a functional component that allows data to be transferred from the corresponding monitored element to the framework. A Data Source is capable to choose the communication method, either polling or pushing, to gather or receive data from the monitored elements.

Polling and Pushing are main approaches used for distributed information dissemination [32,33]. The pull model is based on the request and response paradigm. In other words, the user initiates the data transfer and requests to the server for a specific piece of information, either synchronously or asynchronously. The server receives the request and responses back to the initiator with the requested information. Examples of the pushing approach include SNMP [34] or HTTP [35]. Meanwhile, the pushing method is based on the publish/subscribe/distribute paradigm. In this case, each agent (publisher) individually takes the initiative and sends the information to the subscribers through a distributed engine. The subscribers listen the set of channels based on their individual interest. Examples of pushing approaches include GCM [36], XMPP [37].

A Data Source implements the required interface to communicate with the monitored element. For example, in order to receive data from a particular sensor, the Data Source may implement a server-side REST interface with a particular subset of methods exposed to the sensors, letting them send data to the monitoring framework. The Discovery function is related with the framework capabilities to detect the instance of new data sources and communicate them in order to collect metrics.

### 4.2. Virtual Sensors Descriptor

The Virtual Sensors Descriptor component is responsible to receive, parse and store the information regarding onboarded sensor types available on SELFNET catalogue. This element interacts with two other SELFNET components: VNF Onboarding Sublayer and Data Sources Manager.

The Virtual Sensors Descriptor uses the SAUvo_SAUma interface to allow the communication with the VNF Onboarding Sublayer. This interface uses a communication channel allowing VNF Onboarding Sublayer to report to Virtual Sensors Descriptors component whenever a sensor is onboarded, updated or removed in SELFNET catalogue. Every time an operation is performed in the SELFNET catalogue, the Virtual Sensor Descriptors need to be notified in order to receive this information, parse it and update its internal sensor type database.

The VNF Onboarding Sublayer provides a message queue to broadcast any action performed on the catalogue, the Virtual Sensors Descriptors have a client that subscribes this channel and updates the local sensor catalogue. The local sensor catalogue is available to the Monitor and Discovery framework via Virtual Sensor Descriptors—Data Sources Manager interface. This catalogue mirrors, from the VNF Onboarding sublayer main catalogue, the essential sensor information needed to connect to a sensor and retrieve the sensed data. The catalogue does not have information regarding running sensor instances, but provides data related to the sensor metrics and communication.

### 4.3. Data Sources Manager

In the SELFNET monitoring framework, the Data Source is defined as a functional component in the monitor side, which serves as an interface between the monitor and the monitored element and is in charge of collecting data from the corresponding monitored device. For the instantiation of the data sources, the Data Sources Manager requires specific information (e.g., communication protocol, poll interval) about the sensor, which is obtained during the sensor onboarding phase. As soon as the sensor is instantiated, the Data Sources Manager is notified by the Orchestrator with the relevant instantiation information (e.g., IP address) from the sensor in order to correctly configure the data source. At this stage, the data source is ready to start collecting data.

### 4.4. Data Sources Instances

**Figure 7:**
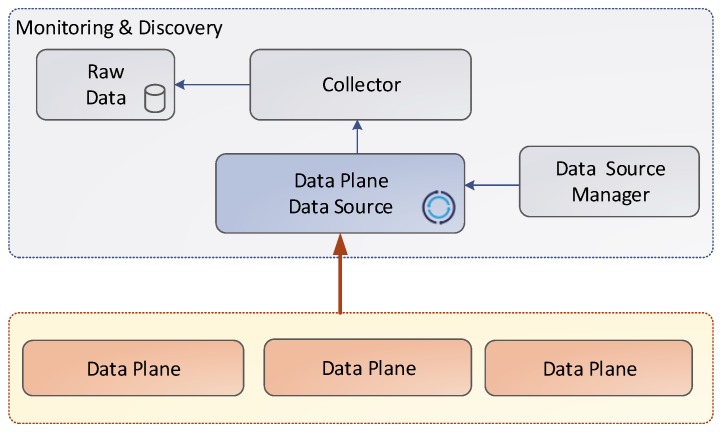
Data Source.

This section describes the different data sources and the way that the metrics are collected. As depicted in the Figure 7, the data collection shares a common structure. The source instances send the metrics to the corresponding data source. Then, the information is stored in the raw data through the collector.

Table 4 summarizes the particular types of metrics depending on the source instances.

### 4.5. Collector

In the SELFNET monitoring framework, the Collector is defined as a functional component in the monitor side, which listens, on a message bus, for Data Sources metrics and events, transform and publish data into a Database and External Consumer Systems. The Collector uses a pluggable storage system, meaning that it can be changed by other databases if needed. This function occurs in multiple pipelines associated with Data Sources in order to provide configurable data flows for transformation and publication of data.

### 4.6. Raw Data Storage

Storing Metrics and Events offers different challenges. Metrics have a regular behaviour and events have an unpredictable nature. SELFNET will use more than one kind of database to store events and metrics. Metrics will be stored in a TSDB (Time Series database). These types of databases are optimized for handling time series data, arrays of numbers indexed by time (a datetime or a datetime range). Events will be stored in a NoSQL database whose main purpose is to store unstructured data for a short period of time. Raw data storage has the main goal to provide a stage where data can be queried for a short period of time. In addition, this storage can be used for more detailed analysis in order to take better decisions. It supports two kinds of policies for metrics that need to be described on sensor descriptors. As an example, policies should look like: 1 day timespan for 1 min granularity and 7 days timespan for 2 min granularity. This time aggregation is not intended to overlap features of the Aggregation Layer, it is only a way to support raw data for longer periods.

### 4.7. Northbound API

The Monitoring and Discovery Framework exposes several APIs for other SELFNET components, but mainly for the Aggregation Framework to retrieve the available data. All stored data is published to a message bus, included in the Monitoring API, and can be reached by other SELFNET consumers. This should be the preferred method for forwarding data to the Aggregation layer.

The information stored in the Raw Database can be accessed by a REST API. This API supports authentication and enable multiple operations: listing all metrics created, retrieving measures and filtering them over a time range, among others. It is also possible to query data for a specific granularity and for all available granularities. Optionally, data related to Events is stored in a Raw Database, but the preferred method to consume it is from message bus without any treatment or association logic. The database also exposes a REST API in order to query events that occurred in a near past.

## 5. Monitoring and Discovery Workflows

This section highlights the workflows across different components of Monitoring Framework and their relation with external components like the Onboarding or Orchestrator Sublayer.

### 5.1. Sensors Onboarding

**Figure 8:**
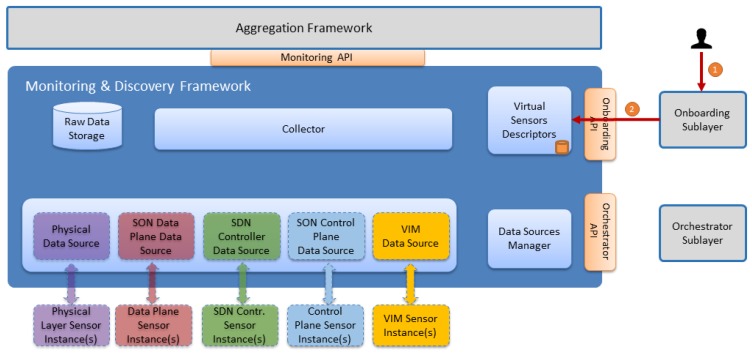
Sensor Onboarding Workflow.

Whenever a new sensor is onboarded to the architecture, several steps are carried out in order to inform other SELFNET components about the sensor functionalities and descriptors. This process is known as sensor onboarding and is depicted in Figure 8.

Two main components participate in this workflow, the Onboarding Sublayer and Virtual Sensors Descriptors, as is described in Table 5.

### 5.2. Sensor Instantiation

**Figure 9:**
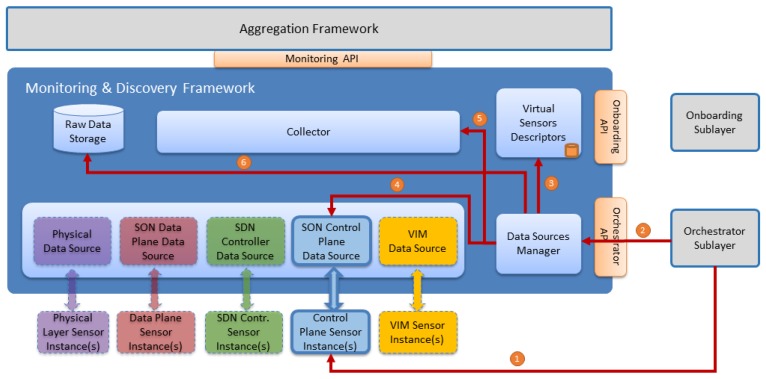
Sensor Instantiation Workflow.

Sensor instantiation has a direct impact in Monitoring and Discovery Framework. Once instantiated, a sensor will start to produce data that will subsequently be available to the Monitoring and Discovery framework. For an effective communication between the sensor and the framework, a valid Data Source is required. Sensor instantiation workflow is depicted in Figure 9.

This workflow is described in Table 6 and it takes into account a Control Plane Sensor Instance. It is important to note that all sensors from the five sources follow the same process.

### 5.3. Sensors Instance Monitoring—Metrics and/or Events Workflow

**Figure 10:**
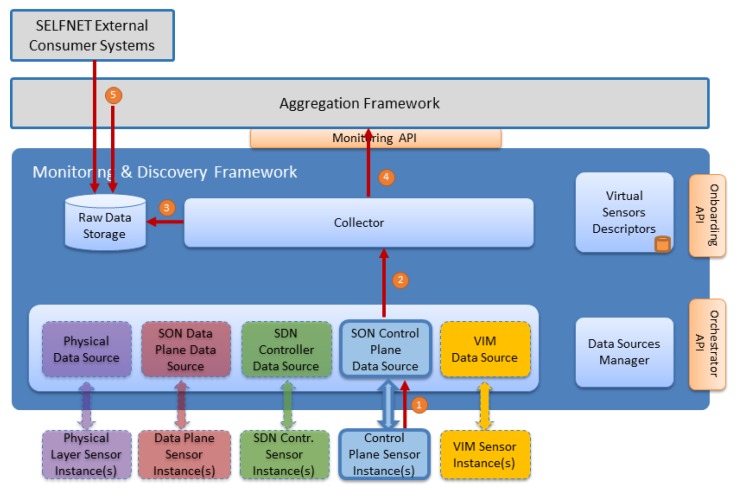
Sensor Monitoring—Metrics and/or Events Workflow.

After sensors are onboarded and instantiated in the virtual environment, data can start flowing, either pushed or polled, to the Monitoring & Discovery Framework. Figure 10 illustrates the sensors data flow (either metrics and/or events) towards the Monitoring & Discovery Framework.

The workflow steps are described in Table 7.

### 5.4. Sensors Monitoring—Data Source Reconfiguration Workflow

**Figure 11:**
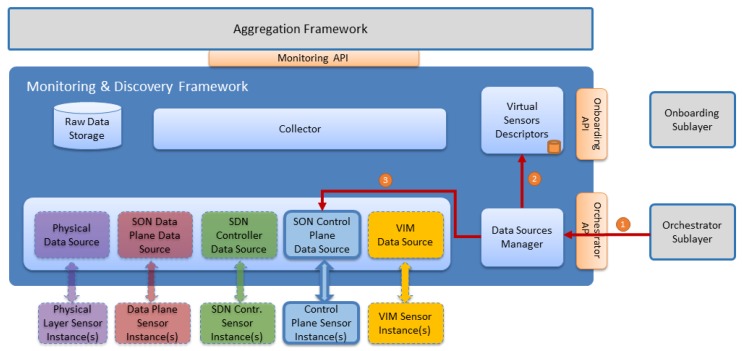
Sensor Monitoring—Data Source Reconfiguration Workflow.

During the sensor instance runtime, some specific parameters can be reconfigured without having to change the sensor instance. For example, one of the parameters that can be changed is the polling period. By default, the polling period is initially defined for each sensor type in the onboarded metadata. However, during the sensor runtime, this parameter can be changed (for example as an autonomic management decision) without affecting the sensor-running instance. This workflow is illustrated in Figure 11.

The detailed steps of this workflow are described in Table 8.

### 5.5. Sensors Instance Removal Workflow

**Figure 12:**
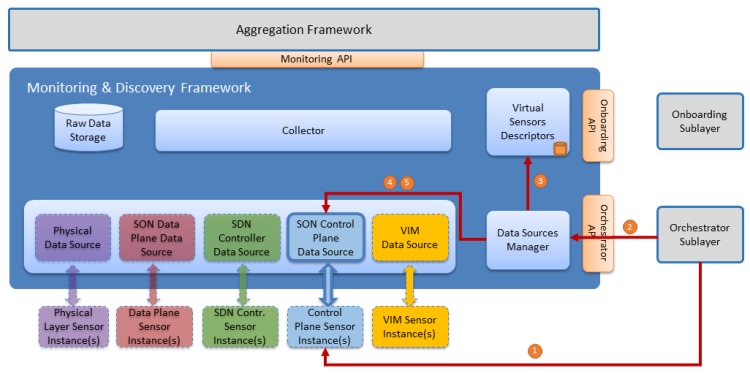
Sensor Instance Removal Workflow.

Finally, when a sensor instance is removed from the infrastructure, the Data Source instance must be removed in the Monitoring and Discovery Framework. This workflow is illustrated in Figure 12.

The workflow steps related with the removal of a sensor instance are described in Table 9.

## 6. Implementation

The prototypal implementation of the Monitoring and Discovery framework has several challenges in order to fulfill the above described requirements. The gathered metrics have several sources depending on the level of collection (physical, virtual, sensor). For this purpose, different open sources have been tested for implementation. Firstly, the Common Monitoring Framework and how each component will interact in the monitoring process were described. Then, the implementation of Virtual Sensors Descriptors component, the implementation of each Data Source, the Data Sources Manager component, the Collector and the Raw Data Storage are detailed.

### 6.1. Common Monitoring Framework

**Figure 13:**
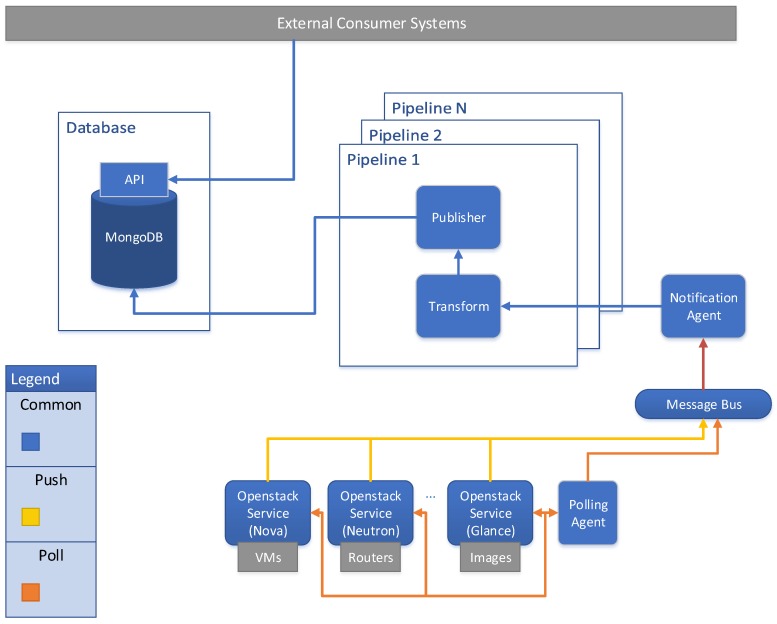
Ceilometer Architecture.

In order to fulfill the Monitoring and Discovery requirements; we have chosen OpenStack project [39,40] and its Telemetry service as the baseline to the framework implementation process. OpenStack appears as a promised open source project to manage cloud platforms through a set of services (nova, neutron, keystone, telemetry, etc.), which could be integrated not only with traditional networks, but also with SDN and NFV approaches. Similarly, the Telemetry Project [41] was developed to facilitate the metering and monitoring of virtual resources from OpenStack deployments. This project manages different branches [42] in order to provide alarming service (Aodh), data collection service (Ceilometer/Monasca) [43] and time-series database and resource indexing service (Gnocchi) [44]. Ceilometer proposes an architecture based on plugins that allows easy scalability, extensibility, meter/alarm customization and tracking of available resources by means of the creation of new agents. A ceilometer agent collects not only information from OpenStack resources, such as Nova or Neutron [45], but also external sources, such as LibreNMS tool [46] and OpenDaylight (ODL) Time Series Data Repository (TSDR) [47]. The Ceilometer architecture is shown in Figure 13.

**Figure 14:**
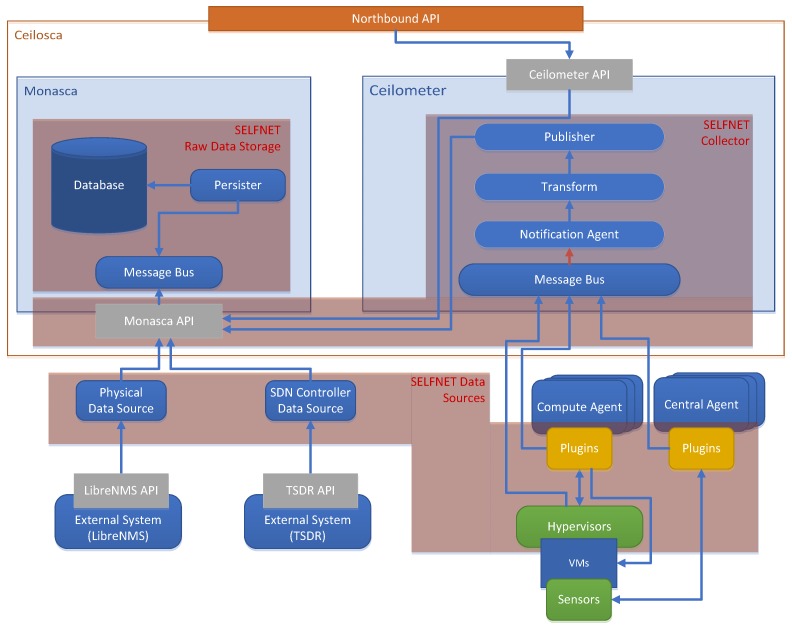
Mapping between Ceilosca and SELFNET Monitoring Framework.

From a metric collector point of view, Ceilometer provides the polling and pushing methods for gathering data. The sources of information include not only OpenStack services, but also additional customizable metrics. However, the increase in the number of sources and resources can decrease exponentially the system performance and cause scalability problems. To address this issue, the Ceilosca project [48] combines Ceilometer (telemetry collector) and Monasca projects [49]. Ceilosca is used as a metric storage tool that provides a fast API for data access. This approach deals with the above mentioned scalability issue [50]. Figure 14 shows the mapping between a SELFNET Monitoring Framework with the Ceilosca approach and how it will operate.

The architecture has the following components:

➢ Data Sources: This component corresponds not only to traditional ceilometer data sources (OpenStack services) but also to new plugins (for sensors to be developed). Data sources are related to the data gathering task. This component is further detailed in Section 6.4.
➢ Collector: This component is composed of the Notification Agent and Ceilometer Pipeline and the Monasca API. This component is further detailed in Section 6.5.
➢ Raw Data Storage: This component storages meters and events. It is related to the Ceilosca database. This component is further detailed in Section 6.6.
➢ Northbound API: It represents the API exposed by the Monitoring and Discovery Framework. It is composed of the Ceilometer API.

### 6.2. Virtual Sensors Descriptors

Virtual Sensors Descriptors is responsible to receive, parse and store the information regarding onboarded sensor types available on SELFNET catalogue (Onborading Sublayer). For this purpose, it defines the next components:

➢ Message Queue client: to listen any action performed on the catalogue.
➢ Local Database: to store the sensor metrics and their kind of communication.
➢ REST server: Virtual Sensor Descriptors—Data Sources Manager interface.

The Monitor and Discovery framework listens for incoming messages provided by the Onboarding Sublayer when a change is made into the catalogue. The REST interface was developed using Python3 and a minimalist Web micro framework named Falcon. Since the sensor metadata is not relational, and it will only exhibit onboarded sensor data, the local database is a MongoDB database, a NoSQL database system. The interface with the VNF Onboarding Sublayer was developed also using Python3 and RabbitMQ, as the Message Bus.

The Virtual Sensors Descriptors expose a REST interface to the Data Sources Manager allowing it to reach the catalogue of sensor information. This interface needs to disclose a single endpoint to retrieve information. The client needs to provide the sensor type identifier in order to receive a JSON response with the information available on the local sensor catalogue. The Message Queue client is prepared to receive three types of actions:

➢ Create—Add a new monitoring piece of metadata to the local database.
➢ Update—Replace the monitoring metadata.
➢ Delete—Delete the stored metadata for a specific sensor.

Each collected metric is composed of a unique name, “metric-name” and a set of sample metrics, each one identified by a name, a resource which provides the origin of the value, a unit and a type (e.g., cumulative or delta). The Virtual Sensor Descriptors also exposes the sensor communication to the Data Sources Manager, composed of the protocol, a set of endpoints with the metric id and the endpoint value, the method (push or poll) and a list with metadata composed of a key value pair.

### 6.3. Data Sources Manager

**Figure 15:**
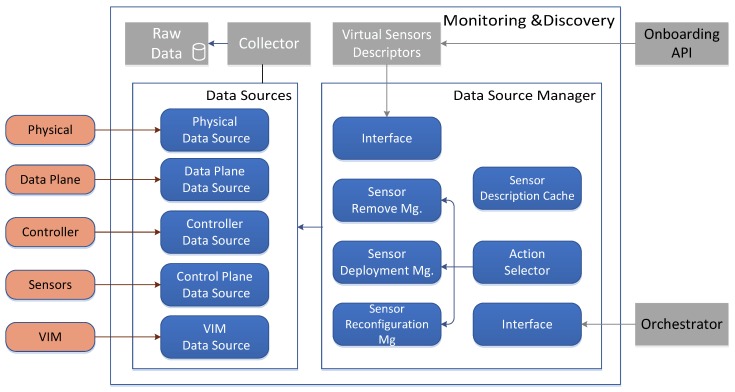
Data Source Manager: Sensor Operations.

To create the corresponding Data Sources in line with the sensor specifications (delivered data, protocol and period), the Data Sources Manager component is implemented. It dynamically creates specific Data Sources to map the type of sensor instantiated in the infrastructure, establishing the communication channel to gather information. In order to receive information about sensor activities (instantiation, reconfiguration, delete), a REST API interface between the Data Sources Manager and the Orchestrator is implemented. Then, the information must be classified by the Action Selector component, as is illustrated in Figure 15. Action Selector is used to analyze the type of action performed by the Orchestrator through specific messages, identifying which operation is done (sensor deployment, remove or reconfiguration). According to the message obtained from the orchestrator, the action selector decides which operation is done and notifies the respective module.

Three functional blocks are implemented: (i) the sensor deployment manager; (ii) the sensor remove manager; and (iii) sensor re-configuration manager. Furthermore, an interface is implemented between Data Sources Manager and Virtual Sensors Descriptors component, which can retrieve the necessary information, such as the type and ID of SDN/NFV applications, the metrics collected by the sensor, the data transmission mode (poll/push), among others. The retrieved information is temporarily stored in the Cache, called Sensor Description Cache. The sensor description is used to manipulate the sensor during the sensor lifecycle.

### 6.4. Data Sources Instances

A Data Source is defined as an interface between the Monitoring Framework and the monitored element and is able to gather data from the corresponding monitored device. For this purpose, the SELFNET framework takes advantage of several open source monitoring tools, as described in Table 10.

### 6.5. Collector

Having in mind the mapping between SELFNET architecture and Ceilosca (Section 6.1). The Collector is composed of the Monasca API and four Ceilometer components: the Message Bus, the Notification Agent, the Transform and the Publisher. The Notification Agent consumes data from the Message Bus, delivering it to the Transform, where the data is normalized. Then the Publisher(s) publish the data into the destination: the Ceilosca database (by using the Monasca API) and external consumer systems (such as a message bus used by the Aggregation Framework). It is important to note that the Transform has more capabilities as is mentioned in Section 6.1. Moreover, the Monasca API can be used to directly publish telemetry data from non-OpenStack services (e.g., LibreNMS, ODL-TSDR) without using Ceilometer.

### 6.6. Raw Data Storage

Ceilometer provides several ways to store both meters and events. By default, Ceilometer stores meters and events in a MongoDB, a NoSQL database, where every record is stored in the database (raw data). As of the date, the Ceilometer project recommends to replace MongoDB by the TDBaaS Gnocchi (Time Series Database as a Service) to store meters, while keeping MongoDB for events storage, especially when facing low latency use cases. The main reason for this change is the scalability problems of MongoDB when stored data are queried (bad performance when increase the amount of records stored). SELFNET Monitoring Framework presents some alternatives to store the data. On one hand, Monasca, by default, stores data using InfluxDB (TDBaaS like Gnocchi). On the other hand, Ceilosca provides a faster and more complete API that enables, not only operations like aggregation, max, count, avg, over data; but also a way to insert non-telemetry data (external to Ceilometer).

### 6.7. Validation–Monitoring GUI

**Figure 16:**
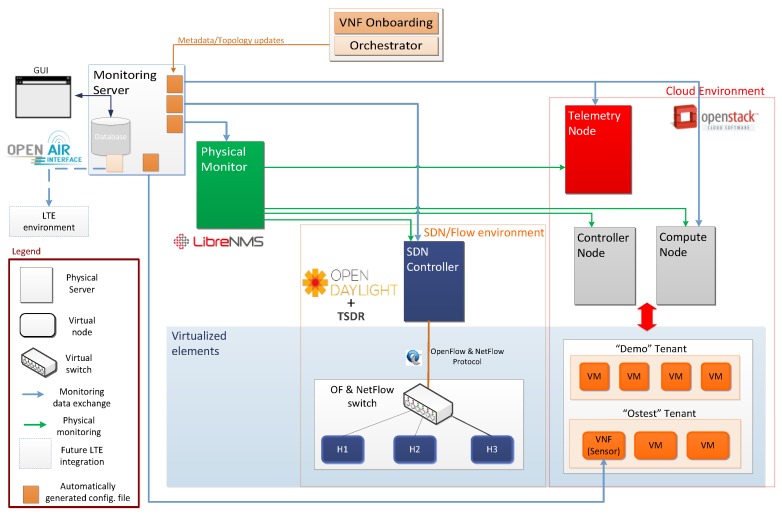
SELFNET Monitoring Framework Testbed.

The Monitoring and Discovery Framework has been implemented in a virtual environment as a proof of concept, over a single physical server (ThinkServer TD350: Intel^®^ Xeon^®^ Serie E5-2600 v3 -16 cores, DDR4 512 GB—2133 MHz). Six virtual machines have been deployed through VirtualBox hypervisor, as is shown in Figure 16. These VMs represent the Controller Node, Compute Node, Telemetry Node, OpenDaylight Node, LibreNMS Node and the Monitoring Server.

The Monitoring Server retrieves the required information from data sources: Physical Monitor (LibreNMS), Cloud Environment (OpenStack), SDN/Flow environment (ODL+TSDR), LTE environment (OpenAir) and Sensors. The Monitoring Server manages the communication not only with the VNF Onboarding Sublayer and the Orchestrator Sublayer, but also with all data sources through a set of configuration files. Physical Monitor is able to collect data from SDN Controller, Compute Node, Telemetry and Controller Node, which emulates a Physical Server. In turn, Telemetry Node collects information from each OpenStack service available in the cloud environment. For its part, Physical Monitor retrieves sensor metrics through the Sensor API. Regarding to the collection of LTE metrics, this is part of the ongoing work. Moreover, the communication between these nodes, are done through two networks, as follows:

(1) The Management Network (MGNT) is in charge of the communication between OpenStack, LibreNMS and ODL Servers.
(2) The Public Network provides internet access and must be reachable by anybody. In particular, some OpenStack API (Neutron, Horizon) are exposed via this network.

Furthermore, two OpenStack services are mainly involved in the communication between virtual instances (VMs); (i) the Nova Service that enables the instantiation of a new virtual machine and (ii) the Neutron Service that is responsible of the management of the virtual networks.

**Figure 17:**
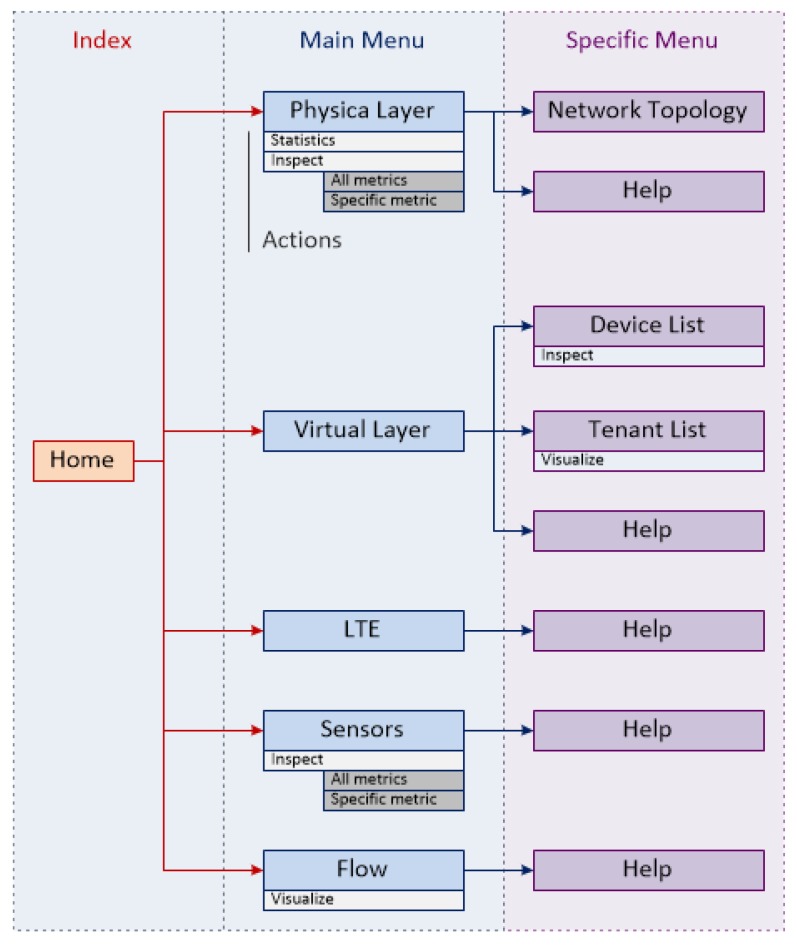
SELFNET Monitoring GUI navigability.

In order to visualize the collected information, a simple Monitoring GUI was developed as a three-tier architecture based on a client-server approach. This GUI shows selected data from physical layer devices, virtual layer devices, LTE, sensors and flows level (Figure 17).

**Figure 18:**
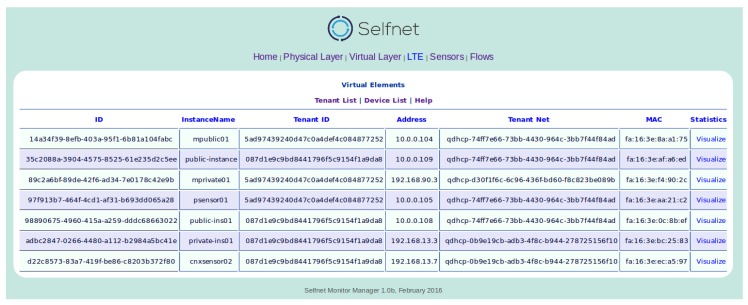
List of SELFNET virtual elements on the Virtual Layer View.

For example, the view of the physical layer displays a list of all discovered network devices and their most relevant parameters: device identification, hostname, location, port and MAC address. The Virtual Layer View shows the tenant list (List of tenant with their respective ID, name and description) and the device list (related information of each virtual instance) as is illustrated in Figure 18.

**Figure 19:**
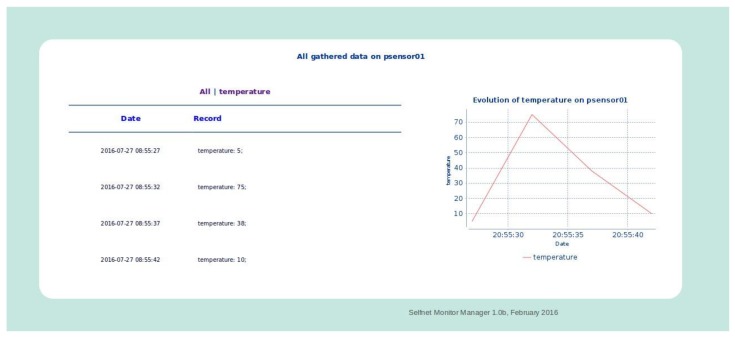
Details of information gathered by a SELFNET Sensor.

For its part, the Flow View describes information about the gathered flow metrics (OpenFlow and NetFlow) and the Sensor View displays a list of all deployed sensors and their most relevant parameters: sensor identification, IP Address, tenant identification, tenant network identification, location, type of sensor, and their values and statistics; as is illustrated in Figure 19.

It is important to note that “psensor01” is a simple temperature sensor. It is running in a virtual machine of a specific tenant (ostest), from which the data is retrieved every 5 s. The monitored metrics are defined in the Onboarding Sublayer and the instantiation information in the Orchestrator Sublayer. This example shows the temperature values for each sample and their evolution over time. This module is able to (i) customize the monitored metrics by each sensor type and (ii) gather these metrics from the respective data source.

An additional way in validating the capabilities of the framework includes the analysis of specific Use Cases. The SELFNET Self-Optimization Use Case [52] improves the end users perceived QoE in video streaming services. In this case, the sensors are deployed in the virtualized infrastructure. Sensors monitor network state metrics, new ultrahigh definition (U-HD) video and energy related metrics. The combination of these low level metrics is combined to address innovative Health of Network (HoN) composite metrics. SELFNET is able to detect and respond when QoE levels either have fallen below or are predicted to fall below expected levels. Similarly, the energy efficiency of the system can be analyzed to proactively managing the energy use of resources across the network. Meanwhile, the SELFNET Self-Healing Use Case [53] is applied to reactively or preventively deal with the detected or predicted network failures. The different metrics collected by the sensors give a global view of the available networking resources and services running on virtualized infrastructure. In this way, the system can be adapted to new load situations through the instantiation of virtual load balancers in strategical points. Additionally, SELFNET is able to use the historical references to predict high-resource demands before they happen and take actions about them.

## 7. Conclusions and Future Work

The design and prototype implementation of the SELFNET Monitoring and Discovery framework were presented in this document. The proposed architecture is aligned with the latest and innovative trends at the ongoing network monitoring research activities and guarantee easy deployment and scalability. The SELFNET Monitoring and Discovery Framework has included the achieved innovations towards monitoring the different sources within 5G Infrastructures. Particularly, the SELFNET Monitoring and Discovery framework will be able to collect and organize monitored information according to different criteria, such as virtual instances by tenant, metrics by virtual instances or physical devices by network location. Moreover, the SELFNET Monitoring facilitates not only to query the gathered information, but also to manage large amounts of data from heterogeneous data sources. The information of each SELFNET layer may be correlated to provide enhanced information of the network status such as the relationship between virtual instances and their respective physical device, the physical and virtual instances where a sensor is running. This information will be available to SELFNET Aggregation and Correlation, and hence to the SELFNET Analysis process.

The future works include the development of the other SELFNET sublayers, such as SON Control Plane (Sensors), Analyzer, Autonomic Management, Orchestrator, VNF Onboarding, among others. In this way, the evaluation of the use cases and the performance metrics definition will be performed. Similarly, the use of correlation and aggregation techniques in order to define new global HoN metrics capable of identifying network failures, risk and the corresponding responses to mitigate the network problems is included. In the same way, the use of the monitoring available information to predict the future network behaviours in order to prevent the QoS/QoE degradation is also an interest topic. For instance, the potential network congestion and service failures in a HD video application can be mitigated through the prediction of an increase of network traffic. The system can automatically install NFV virtual routers to perform dynamic traffic balancing to adapt the operational capacity with the time-varying traffic demands.

## Figures and Tables

**Table 1:** SDN/NFV based management ongoing projects.

Project	Domain	Description	Application Scenario
CROWD [11]	SDN SON	The project aims to bringing density proportional capacity in heterogeneous wireless access networks. Similarly, it focuses on guaranteeing mobile user’s QoE, optimising MAC mechanisms and proportional energy consumption. In this way, it enhances the traffic management in dense wireless networks.	Traffic Management
5G-NORMA [12]	SDN NFV	The project focuses on providing adaptability of a resource in an efficient way. The framework handles fluctuations in traffic demand resulting from heterogeneous and dynamically changing service portfolio. The novel network functions offer resource-efficient support of varying scenarios and help to increase energy-efficiency.	Multi-service scenario Multi-tenancy scenario
MCN [13]	SDN	The project focuses on the enhancement of traffic processing by means of the separation between radio hardware and packet forwarding hardware.	SDN environment
UNIFY [14]	SDN NFV	The project aims to develop an automated, dynamic service creation platform through the creation of a service model and service reaction language. It enables the dynamic and automatic placement of networking, computing and storage components across the infrastructure. Similarly, the orchestrator includes optimization algorithms to ensure optimal placement of elementary service components across the infrastructure.	Infrastructure Virtualization Flexible Service Chaining Network Service Chain Invocation for Providers
T-NOVA [15]	SDN NFV	The project focuses on the deployment on Network Functions-as-a-Service (NFaaS) over virtualised Network/IT infrastructures. For this purpose, it designs and implements a management/orchestration platform for the automated provision, configuration, monitoring and optimization of virtualized resources. Moreover, SDN is also used for efficient management of the network infrastructure.	High-Level Scenario VNF Channing Scenario

**Table 2:** Monitor and Analyzer Interfaces.

Interface Name	Source	Destination	Information
ILphy_SAUma	Physical	Monitor and Analyzer	Physical metrics
OMLvim_SAUma	Virtualized Infrastructure Manager (VIM)	Monitor and Analyzer	Virtual resources and metrics
DNLdp_SAUma	SON Data Plane	Monitor and Analyzer	Data Plane metrics
CLsc_SAUma	SDN Controllers	Monitor and Analyzer	SDN Controller metrics
SAUvo_SAUma	VNF Onboarding	Monitor and Analyzer	NFV Sensors description
SAUor_SAUma	Orchestrator	Monitor and Analyzer	Instantiation of NFV Sensors
SAUma_SAUam	Monitor and Analyzer	Autonomic Management	High level summary of actual and predictive network issues
SAUma_SACbr	Monitor and Analyzer	API Broker	Status of Monitoring and Analyzer

**Table 3:** Framework architectural requirements.

Requirement	Description
Layered architecture	The framework follows a layered architecture, including a number of ordered and logically separated layers. In this way, the complexity on development process (design, implementation, evaluation) is reduced. Similarly, the interoperability between different vendors and technologies is supported.
Extensibility/Flexibility	The different layers and sublayers are composed using a modular design. The framework modularity offers advantages in terms of the usefulness in extensibility/augmentation. The open interfaces and APIs can be optimized by third parties to adapt their own solutions. Similarly, additional functionalities can be permanently or temporally integrated.
Multi-level scalability	The framework addresses network management concerns in large-scale networks. The monitoring sources are spread over virtualized network elements located strategically at regional or global levels. The framework enables elastic scalability employing the cloud computing engine.
Standards compliance	The design of the framework adheres to the standards that are considered relevant to the domain. It includes ETSI standards for NFV [24] and Open Networking Foundation (ONF) standards for SDN [5].

**Table 4:** Data Source Instances.

Data Source Instance	Description
Physical Infrastructure	Metrics related with the physical equipment, which holds the virtual resources. It also includes the network elements that enable connectivity between the different components.
Virtual Infrastructure Manager	Metrics about the virtualized resources from the cloud environment that are running on top of the physical sublayer. It includes the tenants, virtual switching and routing management, and the location of compute resources attached to switch ports.
SDN Controller	It provides the information related with the network behaviour based on the view of the SDN Controller. In other words, the controller can infer the network statistics based on the information sent by the control plane—data plane interface (e.g., OpenFlow).
SON Data Plane	It provides low level network traffic characteristics. In this context, the flow level monitoring has become an appropriate solution. Flow level measurements can provide useful traffic statistics with small amounts of measured data.
SON Control Plane	It provides the collection of metrics from sensors that will be deployed through the entire virtual infrastructure. These sensors measure and collect specific physical and/or virtual metrics depending on the purpose of the sensor.

**Table 5:** Sensor Onboarding Workflow Steps.

Step	Source Component	Destination Component	Description
1	API Broker Sublayer	Onboarding Sublayer	A new virtual sensor is onboarded to the system either by using the SELFNET Graphical User Interface (GUI) or by an external system. Further details about the virtual sensor onboarding process can be found in Deliverable 3.1 [38].
2	Onboarding Sublayer	Virtual Sensors Descriptors	The onboarding of a new sensor is published by the Onboarding Sublayer to a message bus to which the framework is subscribed by means of the Onboarding API. The Virtual Sensors Descriptors takes care of collecting all the information needed to build its own representation of the sensor.

**Table 6:** Sensor Instantiation Workflow Steps.

Step	Source Component	Destination Component	Description
1	Orchestrator Sublayer	Sensor Instance	A new virtual sensor (e.g., Control Plane) is instantiated by the Orchestrator Sublayer (using the VIM).
2	Orchestrator Sublayer	Data Sources Manager	When the sensor is deployed in the virtual infrastructure, the Orchestrator Sublayer sends a notification about the successful instantiation that is received by the Data Sources Manager component. The notification message contains all sensor instantiation information (sensor type, IP address, port, tenant ID, etc.).
3	Data Sources Manager	Virtual Sensors Descriptor	When a new virtual sensor is instantiated, the Data Sources Manager requests the available metadata (full set of attributes related to a specific sensor, including the output parameters, sensing period, sensor type, etc.) from the Virtual Sensors Descriptors component.
4	Data Sources Manager	Data Sources Instances	Once the Data Source Manager receives the instantiated virtual sensor metadata from the Virtual Sensors Descriptor component, it creates and configures a new data source instance (in this example is the SON Control Plane Data Source) to be prepared to start collecting (listening or polling) data from the sensor. Onboarded information (metrics types, events types, communication protocol, poll periods, etc.) and instantiation information (sensor type, IP address, port, tenant ID, etc.) are used by the Data Sources Manager to instantiate and configure the data source.
5	Data Sources Manager	Collector	The Data Sources Manager component provides the Collector component with the metrics and events that will be collected from the new virtual sensor, during the monitoring workflow.
6	Data Sources Manager	Raw Data Storage	Finally, the Data Sources Manager component provides to the Raw Data Storage component the new metrics (from the new virtual sensor) that should be stored and how (granularity of the stored metrics).

**Table 7:** Sensor Monitoring—Push Metrics and/or Events Workflow Steps.

Step	Source Component	Destination Component	Description
1	Sensor Instance	Data Source Instance	Data is provided by the sensor. Two alternative mechanisms are provided: Push and Polling.
2	Data Source Instance	Collector	The Data Source Instance receives the raw metrics and/or events and provides these to the Collector (specifically, to the Collector Message Bus) using a common API.
3	Collector	Raw Data Storage	The Collector component transforms/normalizes the received data and provides it to the Monitoring and Discovery Framework Raw Data Storage component.
4	Collector	Aggregation Framework	As in step 3, besides providing the received data to the Raw Data Storage component, the Collector component also delivers the collected data to the Aggregation Framework (using a message bus).
5	SELFNET external Consumer Systems	Raw Data Storage	Raw data (either metrics and/or events) can be retrieved/consumed by any SELFNET external system component using the exposed Raw Data Storage API.

**Table 8:** Sensor Monitoring—Poll Metrics Workflow Steps.

Step	Source Component	Destination Component	Description
1	Orchestrator Sublayer	Data Sources Manager	Orchestrator sublayer requests the Monitoring and Discovery Framework to modify the sensor instance polling period (e.g., due to an autonomic management decision).
2	Data Sources Manager	Virtual Sensors Descriptors	The Data Sources Manager requests the Virtual Sensors Descriptors for the sensor metadata information (if required).
3	Data Sources Manager	Data Source Instance	The Data Sources Manager requests the running data source instance to reconfigure the new polling period.

**Table 9:** Sensor Instance Removal Workflow Steps.

Step	Source Component	Destination Component	Description
1	Orchestrator Sublayer	Sensor Instance	The Orchestrator Sublayer removes the running sensor instance (e.g., decision from the autonomic management system).
2	Orchestrator Sublayer	Data Sources Manager	The Orchestrator Sublayer notifies the Data Sources Manager that a specific sensor instance was removed.
3	Data Sources Manager	Virtual Sensors Descriptors	The Data Sources Manager requests the Virtual Sensors Descriptors for the sensor metadata information (if required).
4	Data Sources Manager	Data Source Instance	The Data Sources Manager requests the running data source instance to remove the configuration for the removed sensor instance.
5	Data Sources Manager	Data Source Instance	If the removed data sensor instance is the last instance of that data source type, the Data Sources Manager also removes the Data Sources Instance.

**Table 10:** Open Source dependences.

Module	Tool
Physical Infrastructure	LibreNMS [46]
SDN Controller	ODL [51]
SON Data Plane	ODL-TSDR [47]
VIM	OpenStack [40]
Control Plane Sensor	Ceilometer [41]

## Abbreviations

The following abbreviations are used in this manuscript:

BSS	Business Support Systems
DPI	Data Packet Inspection
ETSI	European Telecommunications Standards Institute
HoN	Health of Network
LTE	Long Term Evolution
NF	Network Function
NFV	Network Function Virtualization
NFVI	Network Function Virtualization Infrastructure
NOS	Network Operating System
ODL	OpenDaylight
OSS	Operational Support Systems
QoS	Quality of Service
QoE	Quality of Experience
SDN	Software Defined Networking
SELFNET	Self-Organized Network Management in Virtualized and Software Defined Networks
SLA	Service Level Agreement
SNMP	Simple Network Management Protocol
SON	Self Organizing Networks
TSDB	Time Series Database
VIM	Virtual Infrastructure Manager
VM	Virtual Machine
VN	Virtual Network
